# Tissue classification from raw diffusion‐weighted images using machine learning

**DOI:** 10.1002/mp.17810

**Published:** 2025-04-08

**Authors:** Guangyu Dan, Cui Feng, Zheng Zhong, Kaibao Sun, Ping‐Shou Zhong, Daoyu Hu, Zhen Li, Xiaohong Joe Zhou

**Affiliations:** ^1^ Center for Magnetic Resonance Research University of Illinois Chicago Illinois USA; ^2^ Department of Biomedical Engineering University of Illinois Chicago Illinois USA; ^3^ Department of Radiology Tongji Hospital Huazhong University of Science and Technology Wuhan China; ^4^ Department of Mathematics, Statistics, and Computer Science University of Illinois Chicago Chicago Illinois USA; ^5^ Departments of Radiology and Neurosurgery University of Illinois Chicago Chicago Illinois USA

**Keywords:** cervical cancer detection, cervical cancer staging, diffusion MRI, machine learning, model‐free analysis, Monte‐Carlo simulation

## Abstract

**Background:**

In diffusion‐weighted imaging (DWI), a large collection of diffusion models is available to provide insights into tissue characteristics. However, these models are limited by predefined assumptions and computational challenges, potentially hindering the full extraction of information from the diffusion MR signal.

**Purpose:**

This study aimed at developing a MOdel‐free Diffusion‐wEighted MRI (MODEM) method for tissue differentiation by using a machine learning (ML) algorithm based on raw diffusion images without relying on any specific diffusion model. MODEM has been applied to both simulation data and cervical cancer diffusion images and compared with several diffusion models.

**Methods:**

With Institutional Review Board approval, 54 cervical cancer patients (median age, 52 years; age range, 29–73 years) participated in the study, including 26 in the early FIGO (International Federation of Gynecology and Obstetrics) stage (IB, 16; IIA, 10) and 28 the late stage (IIB, 8; IIIB, 14; IIIC, 1; IVA, 3; IVB, 2). The participants underwent DWI with 17 *b*‐values (0 to 4500 s/mm^2^) at 3 Tesla. Synthetic diffusion MRI signals were also generated using Monte‐Carlo simulation with Gaussian noise doping under varying substrates. MODEM with multilayer perceptron and five diffusion models (mono‐exponential, intra‐voxel incoherent‐motion, diffusion kurtosis imaging, fractional order calculus, and continuous‐time‐random‐walk models) were employed to distinguish different substrates in the simulation data and differentiate different pathological states (i.e., normal vs. cancerous tissue; and early‐stage vs. late‐stage cancers) in the cervical cancer dataset. Accuracy and area under the receiver operating characteristic (ROC) curve were evaluated. Mann–Whitney U‐test was used to compare the area under the curve (AUC) and accuracy values between MODEM and the five diffusion models.

**Results:**

For the simulation dataset, MODEM produced a higher AUC and better accuracy, particularly in scenarios where the noise level exceeded 5%. For the cervical cancer dataset, MODEM yielded the highest AUC and accuracy in cervical cancer detection (AUC, 0.976; accuracy, 91.9%) and cervical cancer staging (AUC, 0.773; accuracy, 69.2%), significantly outperforming any of the diffusion models (*p* < 0.05).

**Conclusions:**

MODEM is useful for cervical cancer detection and staging and offers considerable advantages over analytical diffusion models for tissue characterization.

**Abbreviations**
ADC: apparent diffusion coefficient
AUC: area under the receiver operating characteristic curve
CTRW: continuous‐time random‐walk
DKI: diffusion kurtosis imaging
DL: deep learning
DWI: diffusion‐weighted imaging
EPI: echo‐planar imaging
FIGO: International federation of gynecology and obstetrics
FOV: field of view
FROC: fractional order calculus
IVIM: intravoxel incoherent motion
kNN: k‐nearest neighbor
ML: machine learning
MODEM: model‐free diffusion‐weighted MRI
SD: standard deviation

## 1 INTRODUCTION

For more than three decades, diffusion‐weighted imaging (DWI) has been increasingly used for cancer detection due to its ability to identify lesions that might be overlooked by other MRI sequences.^1^, ^2^ The features of the diffusion‐attenuated magnetic resonance (MR) signal contain valuable information on water diffusion in tissues, thereby revealing the underlying cellular and/or microstructural alterations associated with pathologic changes. Typically, extraction of such information involves fitting the signal attenuation to a mathematical, biophysical, and/or empirical diffusion model such as the mono‐exponential, intravoxel incoherent motion (IVIM),^3^, ^4^ diffusion kurtosis imaging (DKI),^5^ q‐space trajectory imaging,^6^ restriction spectrum imaging,^7^ fractional order calculus (FROC),^8^, ^9^, ^10^, ^11^ and continuous time random‐walk (CTRW) models.^12^, ^13^, ^14^ Subsequently, a correlation of model parameters with tissue microstructural and/or physiologic parameters is performed. While these models offer valuable insights into tissue characteristics, their effectiveness is limited by predefined assumptions, potentially constraining their ability to fully capture the wealth of information embedded in the diffusion MR signal. Moreover, the application of these models often requires the use of traditional computational algorithms, such as nonlinear least squares fitting^15^ and Bayesian fitting.^16^ Notwithstanding the acquisition of highly oversampled q‐space, these algorithms are susceptible to the inherent limitations such as degeneracy, a phenomenon wherein multiple distinct sets of model parameters can produce nearly identical or indistinguishable estimations.^17^

To overcome the limitations inherent to conventional diffusion models, machine learning (ML)‐based approaches^18^, ^19^, ^20^, ^21^ have emerged as a robust tool for microstructure characterization through pattern recognition. In contrast to traditional methods that rely on fitting predefined models to the diffusion‐weighted signals,^22^, ^23^, ^24^, ^25^, ^26^ ML techniques offer a data‐driven approach, enabling direct classification without the need for explicit modeling of diffusion behaviors. While these ML‐based, model‐free techniques have demonstrated efficacy in classifying various tissue types within healthy populations, their application to pathologically confirmed lesions remains largely unexplored. Furthermore, existing studies lack a comparison with traditional diffusion models. As such, the advantages and potential drawbacks of the model‐free and model‐based approaches have not been well established.

In this study, we introduce a MOdel‐free Diffusion‐wEighted MRI (MODEM) technique aimed to differentiate different tissue types using diffusion signal intensities as input to a ML algorithm without relying on any specific diffusion model. We first demonstrate the feasibility of MODEM using synthetic DWI data from Monte‐Carlo simulations. We then apply MODEM to the detection and staging of cervical carcinomas using clinical diffusion‐weighted images. A comprehensive comparison of the performance between MODEM and several diffusion models is also performed.

## 2 MATERIALS AND METHODS

### 2.1 Data preparation

#### 2.1.1 Monte‐Carlo simulation

Monte‐Carlo simulations of the diffusion MRI signals were performed using Camino Diffusion MRI Toolkit (University College London).^27^ In our simulations, tissue microstructures were modeled in the plane normal to a set of parallelly packed, nonoverlapping cylinders whose boundaries mimic permeable membranes. Random walkers were initiated randomly within a 3D substrate. Their subsequent positions were updated based on the rules outline in a previous study.^28^ The phase change of each random walker was calculated with diffusion gradients perpendicular to the cylinder long axes. Synthetic DWI signals were generated by summing the contribution from all random walkers at the echo time.

To simulate varying cell size, cell density, cell size distribution, and cell membrane permeability (which are collectively referred to as cellular properties), we investigated five distinct substrates for each level of the four cellular properties:

1. Small versus large cell size: cell radius = [2, 3, 4, 5, 6] µm versus [7, 8, 9, 10, 11] µm.
2. Low versus high cell density: intracellular volume fraction = [13.5%, 20.2%, 26.9%, 33.6%, 40.4%] versus [47.1%, 53.8%, 60.5%, 67.3%, 74.0%].
3. Small versus large variation in cell sizes: Gamma distribution with (shape parameter *k*, scale parameter *θ*) = [(10, 0.43 × 10^−6^), (9, 0.47 × 10^−6^), (8, 0.53 × 10^−6^), (7, 0.60 × 10^−6^), (6, 0.69 × 10^−6^)] vs. [(5, 0.81 × 10^−6^), (4, 0.99 × 10^−6^), (3, 1.29 × 10^−6^), (2, 1.82 × 10^−6^), (1.5, 2.30 × 10^−6^)]. These (*k*, *θ*) values were chosen such that the intracellular volume fraction of each substrate was kept the same.
4. Low versus high permeability: permeability = [0%, 0.2%, 0.4%, 0.6%, 0.8%] versus [1.0%, 1.2%, 1.4%, 1.6%, 1.8%].

**FIGURE 1:**
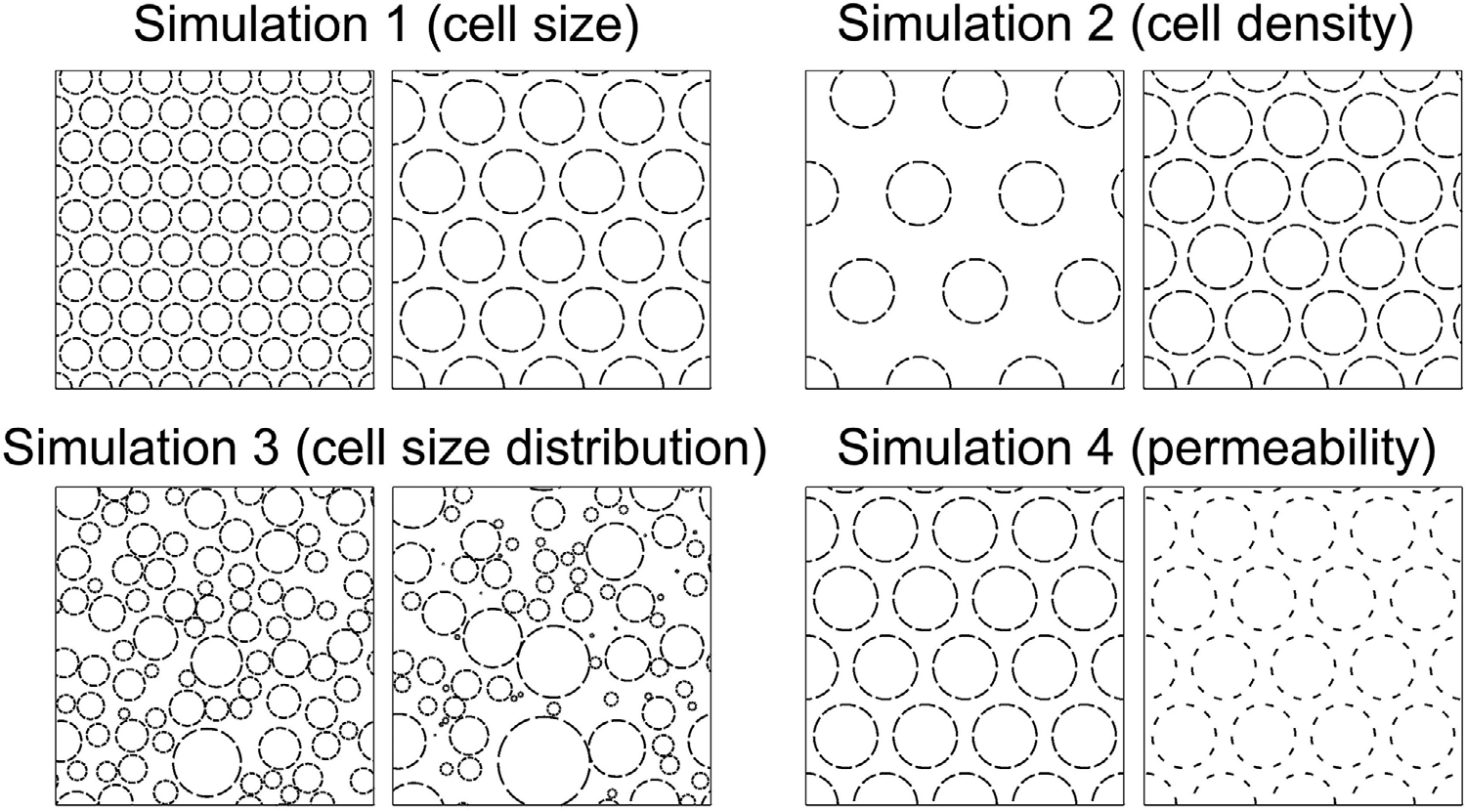
Representative substrates of the Monte‐Carlo simulations. Simulation 1: varying cell size (5 µm vs. 10 µm); simulation 2: varying cell density (33.6% vs. 74.0%); simulation 3: varying cell size distribution (*k* = 10, *θ* = 0.43 × 10^−6 ^vs. *k* = 1.5, *θ* = 2.3 × 10^−6^); simulation 4: varying cell membrane permeability (0.6% vs. 1.2%).

Note that the five numbers in each bracket represent different levels of the corresponding cellular properties. Figure 1 shows a representative example of each group. All simulations employed a common set of parameters (intracellular volume fraction = 58.0%, uniform cell size distribution, hexagonally packed, and permeability = 0.2%, unless specified otherwise), together with the individualized parameters for each substrate out of forty (i.e., 4 cellular properties multiplied by 10 levels each, as specified above. Simulations were performed on an 80‐core Intel Xeon Gold 6230 CPU. In each substrate, 100 000 random walkers were used, each taking 20 000 time‐steps toward the echo time with an intrinsic diffusivity of 2.0 × 10^−3^ mm^2^/s. DWI signals were simulated at each of the13 *b*‐values (0, 50, 100, 150, 200, 500, 800, 1000, 1200, 1500, 2000, 2500, 3000 mm^2^/s). To simulate the Rician noise distribution in the magnitude MRI data, complex Gaussian noise was introduced to the DWI signals at seven noise levels (0%, 1%, 5%, 10%, 15%, 20%, 25%), defined as the noise's standard deviation (SD) relative to the DWI signal amplitude at *b*‐value = 0 s/mm^2^ using a similar approach as described in Barbieri et al.^23^ Simulations in each substrate were repeated 10 times with different random walker initializations, and the noise doping procedures were repeated 1000 times for each simulation, resulting in 10 000 × 13 simulated signals for each substrate at each noise level.

#### 2.1.2 Cervical cancer data

With the approval of the Institutional Review Board of Tongji Hospital and the written informed consent from all participating subjects, a total of 54 cervical cancer patients (median age, 52 years; age range, 29–73 years) were enrolled in this study. All patients were evaluated using FIGO (International Federation of Gynecology and Obstetrics) criteria with the raw T2‐weighted images, diffusion‐weighted (DW) images (*b*‐value = 1000 s/mm^2^), apparent diffusion coefficient (ADC) maps (calculated from *b*‐value of 0 and 1000 s/mm^2^ images), and dynamic contrast‐enhanced (DCE) images. This assessment was conducted by two radiologists (C.F. and D.H.) and subsequently confirmed by histopathological analysis. Among the 54 patients, 26 were in the early FIGO stage (IB, 16; IIA, 10) and 28 were in the late stage (IIB, 8; IIIB, 14; IIIC, 1; IVA, 3; IVB, 2). The clinical characteristics are shown in Table 1. All patients underwent MRI on a 3T scanner (Discovery MR 750; GE Healthcare, Waukesha, Wisconsin) in the supine position with a 32‐channel phased‐array torso coil.

**TABLE 1:** Clinical characteristics of the participants.

Variables	Characteristics
Age (years)^*^	51 ± 10 (29–73)
FIGO stage	
IB	16 (29.6%)
IIA	10 (18.5%)
IIB	8 (14.8%)
IIIB	14 (25.9%)
IIIC	1 (1.9%)
IVA	3 (5.6%)
IVB	2 (3.7%)
Histological subtype	
Squamous carcinoma	47 (87.0%)
Adenocarcinoma	7 (13.0%)
Histologic grade	
Poorly differentiated	15 (27.8%)
Moderately differentiated	10 (18.5%)
Undefined	29 (53.7%)

*Note*: Numbers in parentheses are percentages except where otherwise indicated.

Abbreviation: FIGO, International Federation of Gynecology and Obstetrics.

* Data are means ± standard deviation, with the range in parentheses.

Axial diffusion‐weighted images were obtained using a single‐shot spin‐echo echo‐planar imaging (SE‐EPI) sequence with 17 *b*‐values (0_1_, 50_1_, 80_1_, 100_1_, 150_1_, 200_1_, 300_2_, 500_2_, 800_2_, 1000_4_, 1300_4_, 1700_6_, 2400_6_, 3000_8_, 3600_10_, 4000_12_, and 4500_12_ s/mm^2^), where the subscript denotes the number of averages (NEX). At each nonzero *b*‐value, trace‐weighted images were acquired by successively applying a Stejskal–Tanner diffusion gradient pair along each of the three orthogonal directions to minimize the impact of diffusion anisotropy. Other key acquisition parameters were: repetition time (TR) / echo time (TE) = 2500/85 ms, field of view (FOV) = 40 × 40 cm^2^, matrix size = 128 × 160, slice thickness = 4 mm, slice gap = 1 mm, and the total scan time = 9 min and 17 s.

Regions of interest (ROIs) over the lesion and normal tissues were delineated based on diffusion‐weighted images with a *b*‐value of 1000 s/mm^2^, using T2‐weighted images as a reference. Lesions were outlined along the tumor border with the largest high‐signal area, while normal tissues with low signal intensity were delineated in the area of stroma or myometrium layer adjacent to lesions. This operation was performed by a radiologist (C.F.) with 10 years of experience in female pelvic cancer diagnosis and confirmed by a senior radiologist (D.H.) with over 30 years of experience. Voxels closest to each ROI contour were excluded to reduce the influence of partial volume effects, resulting in 9345 normal tissue voxels and 9835 cancerous tissue voxels. Among all cancerous tissue voxels, 3169 voxels were in patients with early‐stage tumors and 6666 voxels were in patients with late‐stage tumors.

### 2.2 MODEM using ML

In MODEM, we treated DWI signal acquired from each image voxel as a data sample. The data samples in the 80% partition were utilized for training and validation, whereas the remaining 20% partition served as the testing set. To balance the training dataset, a Synthetic Minority Oversampling TEchnique (SMOTE) was applied, which involved synthesizing more data in the less prevalent category (e.g., normal tissue voxels in the cervical cancer detection dataset and early‐stage tumor voxels in the cervical cancer staging dataset).

Feed‐forward backward‐propagation deep neural networks were built using a multi‐layer perceptron classifier (MLPClassifier) in Python Scikit‐learn (version 1.2.2, scikit‐learn.org) to distinguish between different substrates in each of the four substrate pairs (i.e., small vs. large cell size, low vs. high cell density, nonuniform vs. uniform cell size distribution, and less permeable vs. more permeable cell membrane) in the simulation dataset. The same method was subsequently applied to the cervical cancer diffusion‐weighted images with different pathological characteristics (i.e., normal vs. cancerous tissues; and early stage vs. late stage cancers). Each network was comprised of one input layer, six hidden layers, and one output layer. The input layer consisted of *n* neurons (note that *n* is equal to the number of nonzero *b*‐values), where each neuron took one of the normalized DWI signal *S*(*b*)/*S_0_* at a specific nonzero *b*‐value. The six hidden layers, each with *n* neurons, are fully connected with a rectified linear unit activation function. The output layer consisted of two neurons, representing the predicted probability of each class. Training employed the Adam optimizer to minimize the cross‐entropy loss with a maximum of 100 training epochs. A 10% hold‐out validation approach was employed to prevent overfitting. The training process was automatically terminated using early stopping, a regularization technique that halts training after 10 epochs where the loss function evaluated on the validation data exhibited no improvement.

### 2.3 Diffusion model analysis

To compare the performance between MODEM and diffusion models for tissue clarification, the following analytical diffusion models were used:

*Mono‐exponential model*:

(1) $$\frac{S\left(b\right)}{S_{0}}=\exp\left(-bADC\right)\;,$$

where *S*(*b*) is the signal intensity at *b*, *S_0_* is the signal intensity without diffusion weighting, and ADC is the apparent diffusion coefficient.

*IVIM model* ^3^, ^4^:

(2) $$\frac{S\left(b\right)}{S_{0}}=f\exp\left(-bD_{perf}\right)+\left(1-f\right)\exp\left(-bD_{diff}\right),$$

where *D_diff_* is the diffusion coefficient, *D_perf_* is pseudo‐perfusion coefficient, and *f* is pseudo‐perfusion fraction.

*DKI model* ^5^:

(3) $$\frac{S\left(b\right)}{S_{0}}=\exp\left(-bD+\frac{1}{6}\;b^{2}D^{2}K\right),$$

where *D* and *K* are the diffusion coefficient and kurtosis, respectively.

*FROC model* ^8^, ^9^, ^10^, ^11^:

(4) $$\begin{array}{c} \frac{S\left(b\right)}{S_{0}}=\exp\left[-D\mu^{2\left(\beta -1\right)}{\left(\gamma G_{d}\delta \right)}^{2\beta }\left(\Delta -\frac{2\beta -1}{2\beta +1}\delta \right)\right]\;, \end{array}$$

where *G_d_*, *δ*, and Δ are the diffusion gradient amplitude, pulse width, and lobe separation (or diffusion time), respectively. *D* is the diffusion coefficient, *β* is the spatial fractional order derivative, and *μ* is a spatial parameter measured in micrometers.

*CTRW model* ^12^, ^29^:

(5) $$\frac{S\left(b\right)}{S_{0}}=E_{\alpha }\;\left(-{\left(bD_{m}\right)}^{\beta }\right),$$

where *D_m_* is the anomalous diffusion coefficient, and *α* and *β* are the temporal and spatial fractional order derivatives, respectively.

The mono‐exponential, DKI, FROC, and CTRW models were used to fit to the simulation dataset and the cervical cancer dataset. In addition, the IVIM model was also employed to fit to only the cervical cancer dataset because vascularity perfusion was not considered in the Monte‐Carlo simulation. ADC in the mono‐exponential model was estimated from Equation (1) with two *b*‐values of 0 and 1000 s/mm^2^. The IVIM model employed a two‐step approach.^30^ First, diffusion‐weighted signal decay at *b*‐values between 200 and 1000 s/mm^2^ was used to estimate *D_diff_* and *f*. Second, with fixed values of *Ddiff* and *f*, the full bi‐exponential model was used to fit the data at *b*‐values ≤ 200 s/mm^2^ to determine *D_perf_*. For all other models, an iterative Levenberg–Marquardt algorithm was applied to DWI data at all available *b*‐values. All diffusion model analysis was performed on a MATLAB platform (MathWorks, Inc, Natick, MA; Version R2021a).

### 2.4 Statistical analysis

A receiver operating characteristic (ROC) analysis was conducted to assess the diagnostic performance of MODEM and diffusion models on both the simulation and the cervical cancer datasets. For each diffusion model, logistic regression was performed to produce the predicted class probability using all parameters in each model where regression coefficients were estimated using the training data. The mean normalized logistic regression coefficients for cervical cancer detection and staging, computed over 100 iterations, were obtained. Logistic regression coefficients were then scaled by the feature SD and normalized to a sum of 1 within each model. Area under the curve (AUC) and accuracy of diffusion models and MODEM were subsequently obtained on the testing data.

To mitigate sampling bias, the processes of data splitting, classification, and evaluation steps were iterated 100 times with varying random seeds for data split. The mean and SD values of AUC and accuracy for MODEM and diffusion models were then calculated based on the values obtained from 100 iterations. AUC and accuracy values of each diffusion model were compared with MODEM using a Mann–Whitney U‐test.

## 3 RESULTS

### 3.1 Simulation results

**FIGURE 2:**
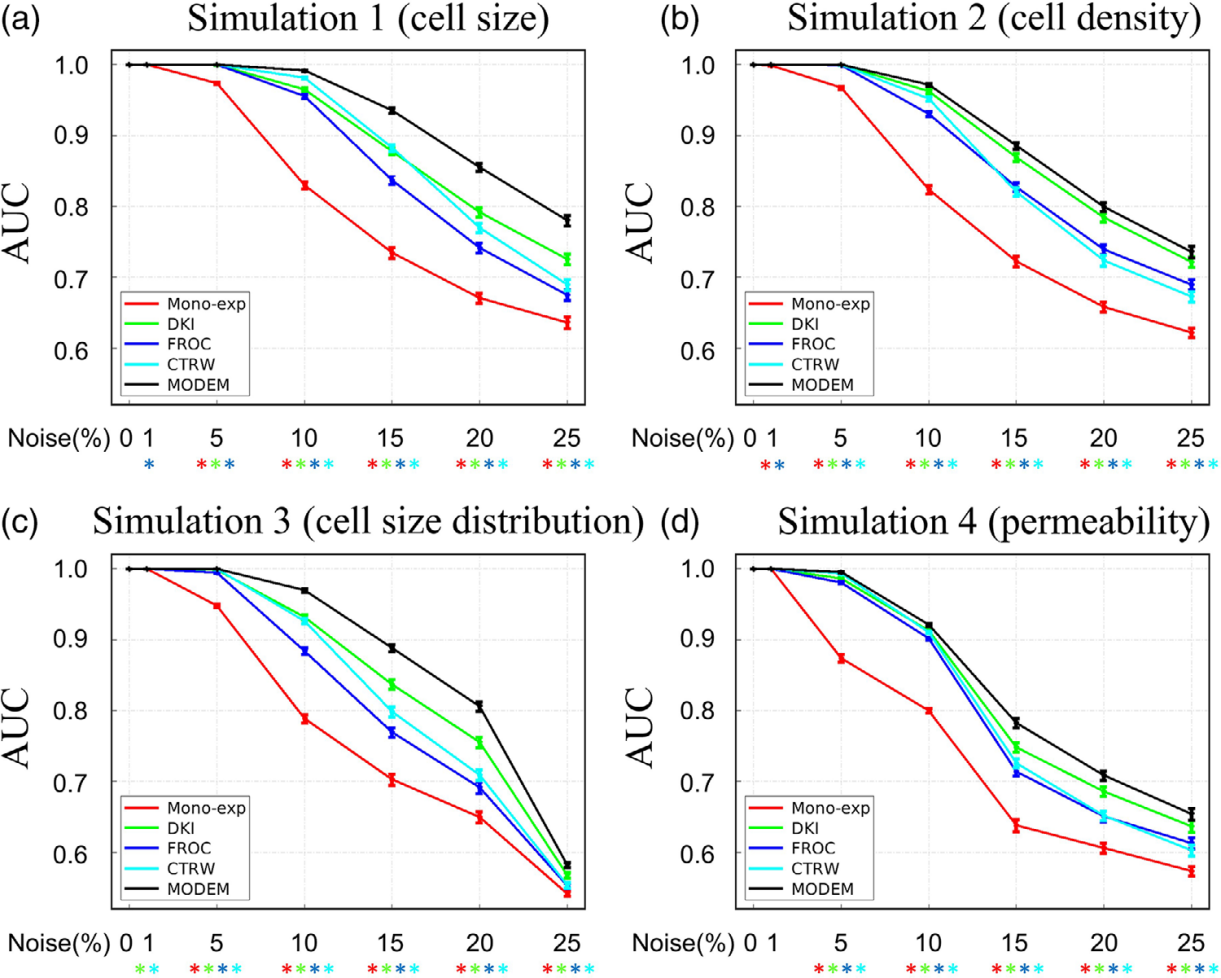
AUC of MODEM and different diffusion models for differentiating substrates in simulation 1: small versus large cell size (a), simulation 2: low versus high cell density (b), simulation 3: nonuniform versus uniform cell size distribution (c), and simulation 4: less permeable versus more permeable cell membrane (d) as a function of different noise levels. The error bars indicate the SD of AUC over 100 iterations. *Indicates statistically significant difference between a given diffusion model and MODEM using *p* < 0.05 as a threshold. Red: mono‐exponential model; green: DKI model; blue: FROC model; and cyan: CTRW model. AUC, area under the curve; CTRW, continuous time random‐walk; DKI, diffusion kurtosis imaging; FROC, fractional order calculus; MODEM, MOdel‐free Diffusion‐wEighted MR; SD, standard deviation.

**FIGURE 3:**
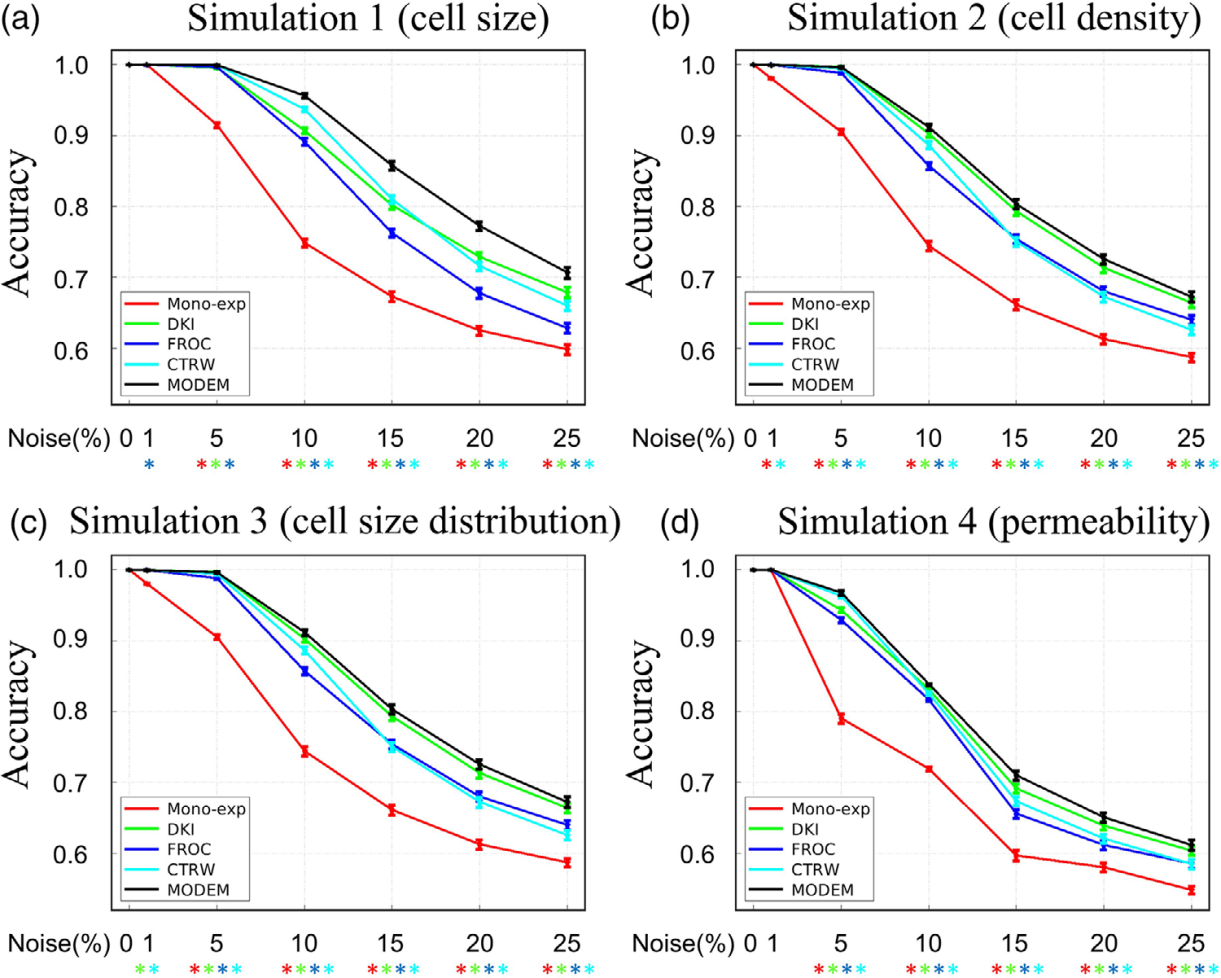
Accuracy of MODEM and diffusion models in differentiating substrates in simulation 1: small vs. large cell size (a), simulation 2: low vs. high cell density (b), simulation 3: non‐uniform vs. uniform cell size distribution (c), and simulation 4: less permeable vs. more permeable cell membrane (d) as a function of different noise levels. The error bars indicate the SD of accuracy over 100 iterations. *Indicates statistically significant difference between a given diffusion model and MODEM using *p* < 0.05 as a threshold. Red: mono‐exponential model; green: DKI model; blue: FROC model; and cyan: CTRW model. AUC, area under the curve; CTRW, continuous time random‐walk; DKI, diffusion kurtosis imaging; FROC, fractional order calculus; MODEM, MOdel‐free Diffusion‐wEighted MR; SD, standard deviation.

Figures 2 and 3 display the results obtained from the simulated datasets. The AUC and accuracy are compared between MODEM and diffusion models for differentiating substrates in the four simulation groups (small vs. large cell size, low vs. high cell density, small vs. large variation in cell sizes, and low vs. high permeability) over 100 iterations at different noise levels. Both the AUC and accuracy values approached 100% in MODEM and all diffusion models when the noise levels were low (≤1%). As the noise level elevated, the AUC and accuracy decreased, as expected. MODEM provided a higher AUC and accuracy over any of the investigated diffusion models when the noise is present. At a noise level of 1% or 5%, statistically significant differences in diagnostic performance were observed between MODEM and part of the diffusion models (see the specific diffusion models and the specific simulation scenarios in Figures 2 and 3). When the noise level is ≥ 10%, statistically significant differences were detected between MODEM and any of the diffusion models investigated. Both the AUC and accuracy of MODEM exhibited excellent stability with SDs within 1% in all simulation scenarios.

Among all diffusion models, DKI exhibited a higher AUC and accuracy than other models in the varying cell density and varying cell permeability simulations at all noise levels, in the varying permeability experiment at noise levels ≥ 10%, and in the varying cell size experiment at noise levels > 15%. Mono‐exponential model yielded the lowest performance metrics in all simulations at all noise levels.

### 3.2 Cervical cancer detection results

**FIGURE 4:**
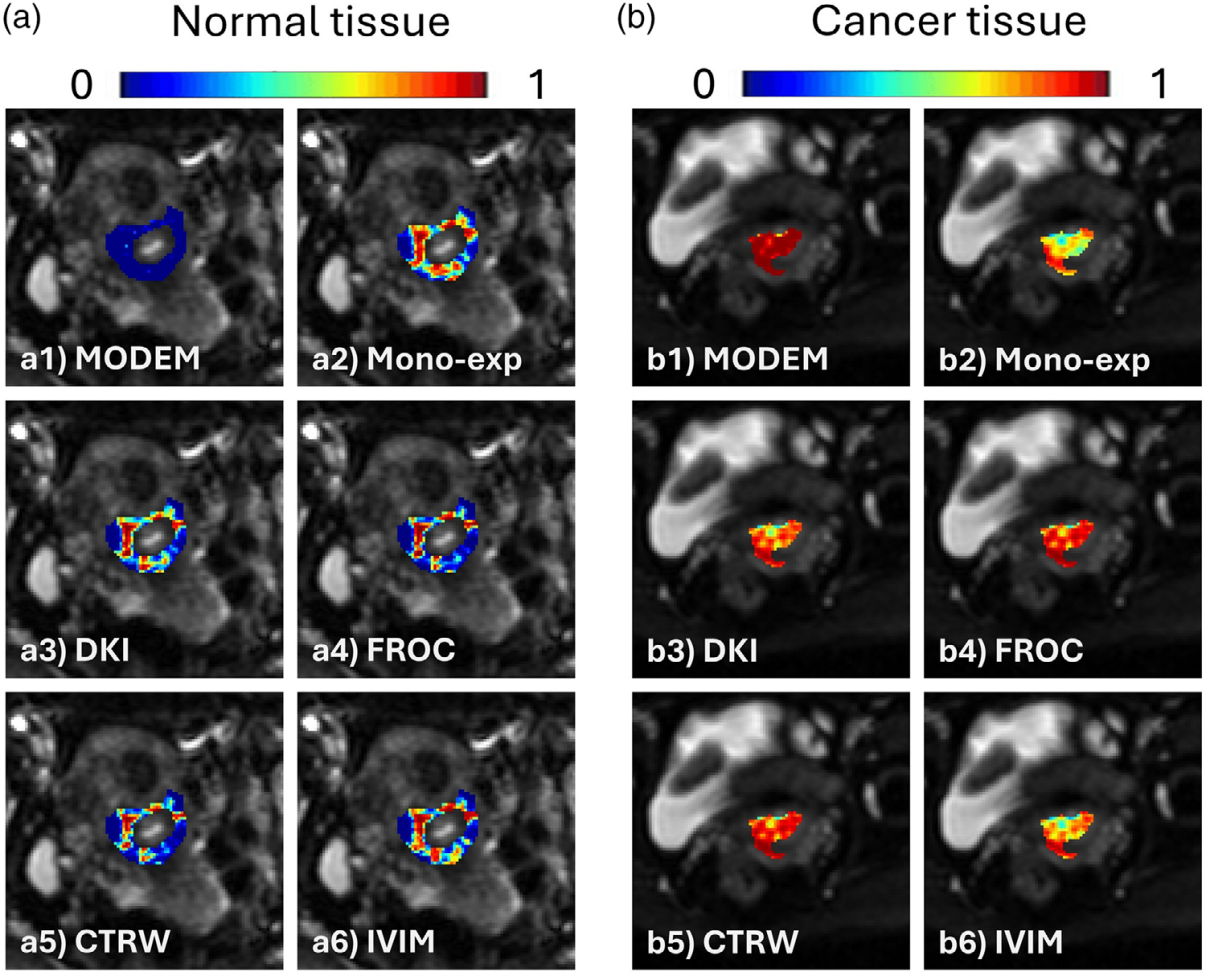
Color‐coded predicted probability map overlaid on DW images of a representative normal tissue ROI from one patient (a) and a cervical cancer tissue ROI from another patient (b). The MODEM results were obtained with one iteration. The color scale represented the probability of the corresponding voxel being classified as cancerous tissue from 0 (blue) to 1 (red) predicted by MODEM (a1, b1), the mono‐exponential model (a2, b2), DKI model (a3, b3), FROC model (a4, b4), CTRW model (a5, b5), and IVIM model (a6, b6). CTRW, continuous time random‐walk; DKI, diffusion kurtosis imaging; FROC, fractional order calculus; IVIM, intravoxel incoherent motion; MODEM, MOdel‐free Diffusion‐wEighted MRI.

**FIGURE 5:**
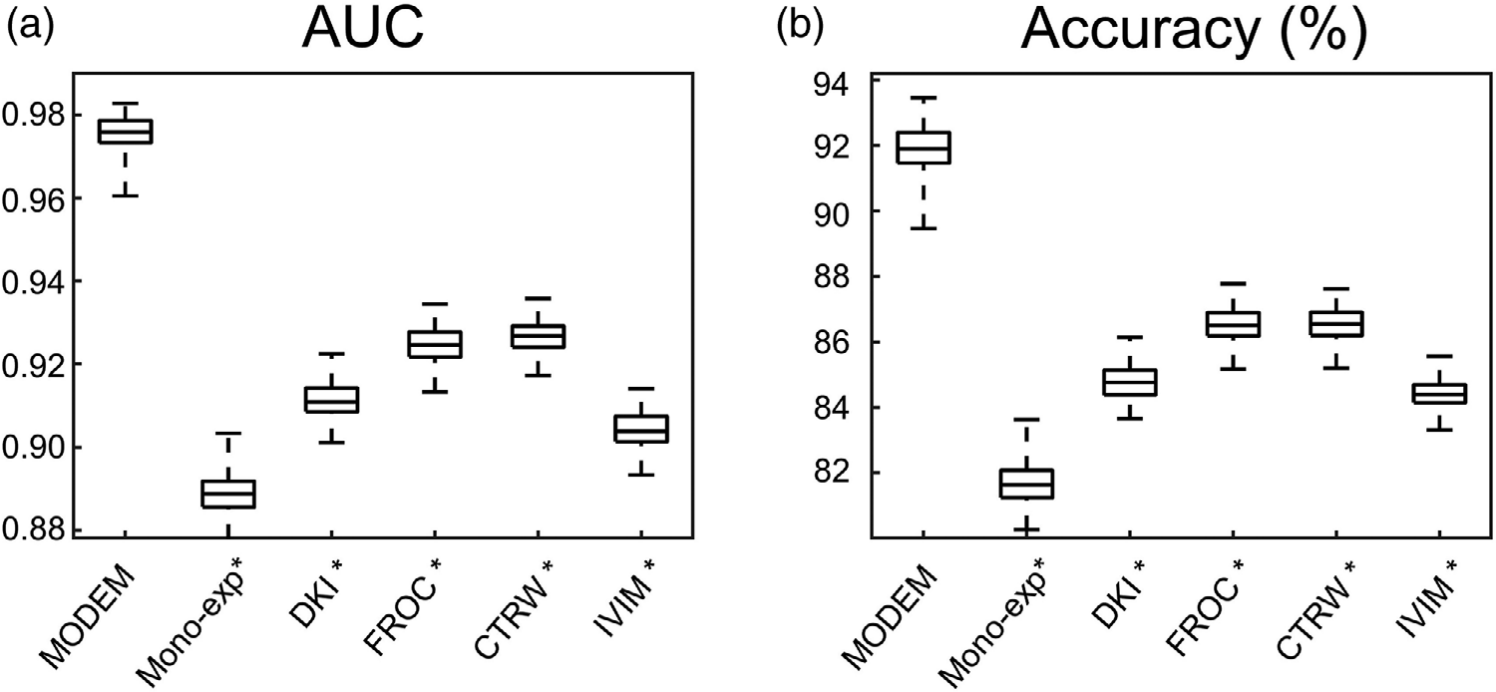
Boxplots of the AUC (a) and accuracy (b) of MODEM and the diffusion models for differentiating cancerous from normal cervical tissues over 100 iterations. *Indicates statistically significant difference between MODEM and a specific diffusion model listed along the horizontal axis using *p* < 0.05 as a threshold. AUC, area under the curve; MODEM, MOdel‐free Diffusion‐wEighted MRI.

Figure 4 shows the color‐coded predicted probability maps from MODEM (with one iteration) and the five diffusion models, each overlaid on DW images of a normal tissue ROI (Figure 4a) or a cancerous tissue ROI (Figure 4b). In the normal tissue ROI, MODEM provided a substantially lower probability that voxels would be classified as cancerous tissue than any of the diffusion models (MODEM, 0.007 ± 0.024; mono‐exponential, 0.451 ± 0.319; DKI, 0.381 ± 0.322; FROC, 0.308 ± 0.332; CTRW, 0.284 ± 0.305; IVIM, 0.409 ± 0.333). In the cancerous tissue ROI, on the other hand, MODEM provided a higher probability that voxels would be classified as cancerous tissue than any of the diffusion models (MODEM, 0.963 ± 0.052; mono‐exponential, 0.656 ± 0.139; DKI, 0.777 ± 0.126; FROC, 0.846 ± 0.114; CTRW, 0.848 ± 0.113; IVIM, 0.717 ± 0.145). The boxplots in Figure 5 compare the AUC and accuracy between MODEM (with 100 iterations) and the diffusion models for differentiating normal versus cervical cancer tissue voxels. Based on the statistical analyses described in Section 2.4, MODEM exhibited a significantly higher (*p* < 0.05) mean AUC (0.976) than any of the diffusion models (0.889 in mono‐exponential, 0.911 in DKI, 0.925 in FROC, 0.927 in CTRW, and 0.904 in IVIM). Similarly, it also produced a higher accuracy (91.9%) than the mono‐exponential (81.7%), DKI (84.8%), FROC (86.5%), CTRW (86.6%), and IVIM (84.4%) models. Table 2 shows that the diffusion coefficient exhibited the highest normalized regression coefficient across most models, indicating its predominant contribution to classification. An exception was observed in the IVIM model, where *D_diff_* and *D_perf_* showed comparable contributions (0.476 and 0.509, respectively).

**TABLE 2:** Normalized logistic regression coefficients in cervical cancer detection and staging.

Model	Parameter	Cervical cancer detection	Cervical cancer staging
Mono‐exp	ADC	1	1
**IVIM**	***D_diff_***	**0.476**	**0.561**
	***D_perf_***	**0.509**	**0.293**
	***f***	**0.015**	**0.146**
**DKI**	***D***	**0.849**	**0.723**
	***K***	**0.151**	**0.277**
**FROC**	***D***	**0.641**	**0.696**
	***β***	**0.319**	**0.288**
	***μ***	**0.040**	**0.016**
**CTRW**	***D_m_***	**0.535**	**0.427**
	***α***	**0.209**	**0.207**
	***β***	**0.256**	**0.306**

Abbreviations: CTRW, continuous time random‐walk; DKI, diffusion kurtosis imaging; FROC, fractional order calculus; IVIM, intravoxel incoherent motion.

### 3.3 Cervical cancer staging results

**FIGURE 6:**
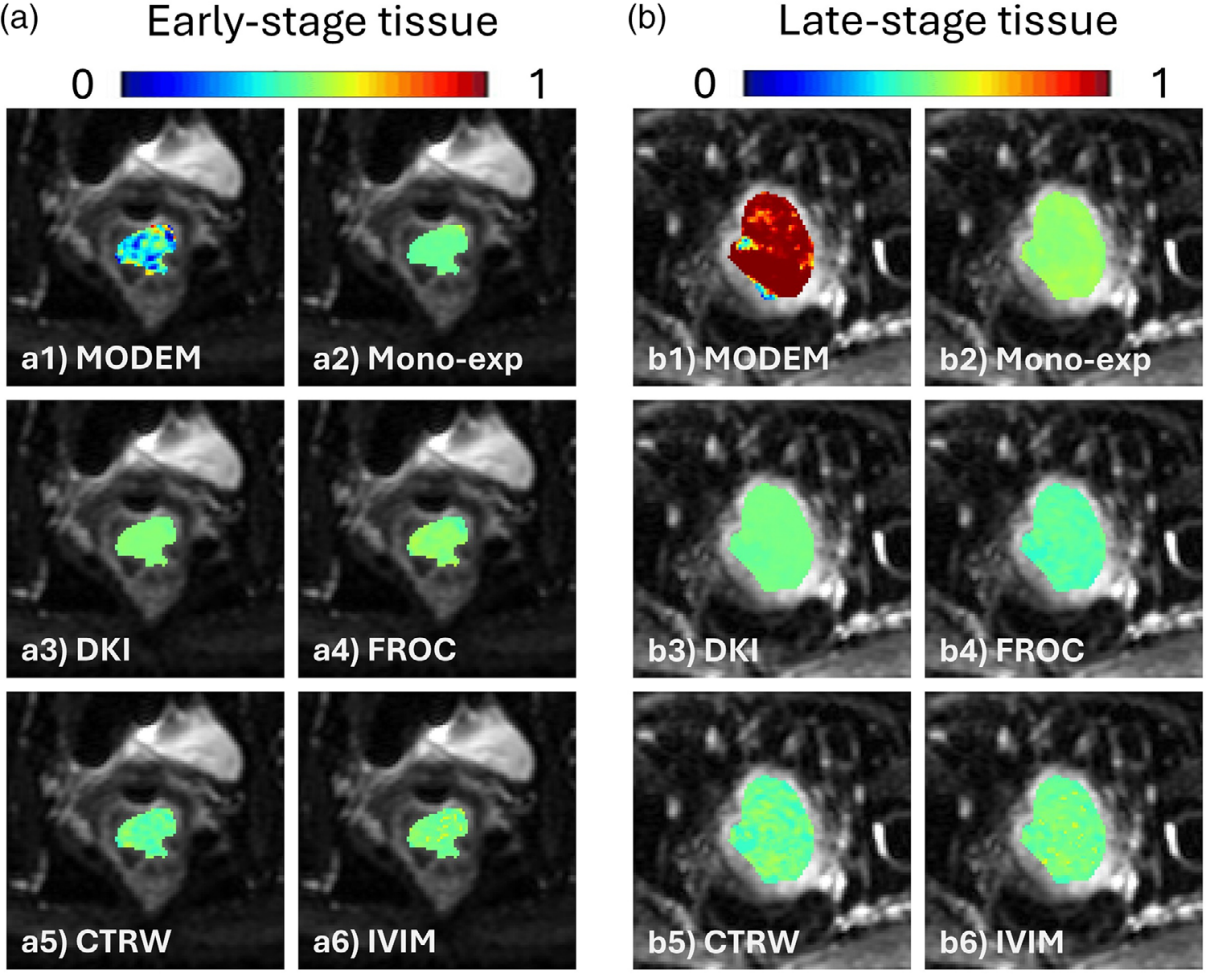
Color‐coded predicted probability maps overlaid on DW images of a representative patient with early‐stage cervical cancer (a) and another patient with late‐stage cervical cancer (b). The MODEM results were obtained with one iteration. The color scale represented the probability of the corresponding voxel being classified as late‐stage cervical cancer from 0 (blue) to 1 (red) predicted by MODEM (a1, b1), the mono‐exponential model (a2, b2), DKI model (a3, b3), FROC model (a4, b4), CTRW model (a5, b5), and IVIM model (a6, b6). CTRW, continuous time random‐walk; DKI, diffusion kurtosis imaging; FROC, fractional order calculus; IVIM, intravoxel incoherent motion; MODEM, MOdel‐free Diffusion‐wEighted MRI.

**FIGURE 7:**
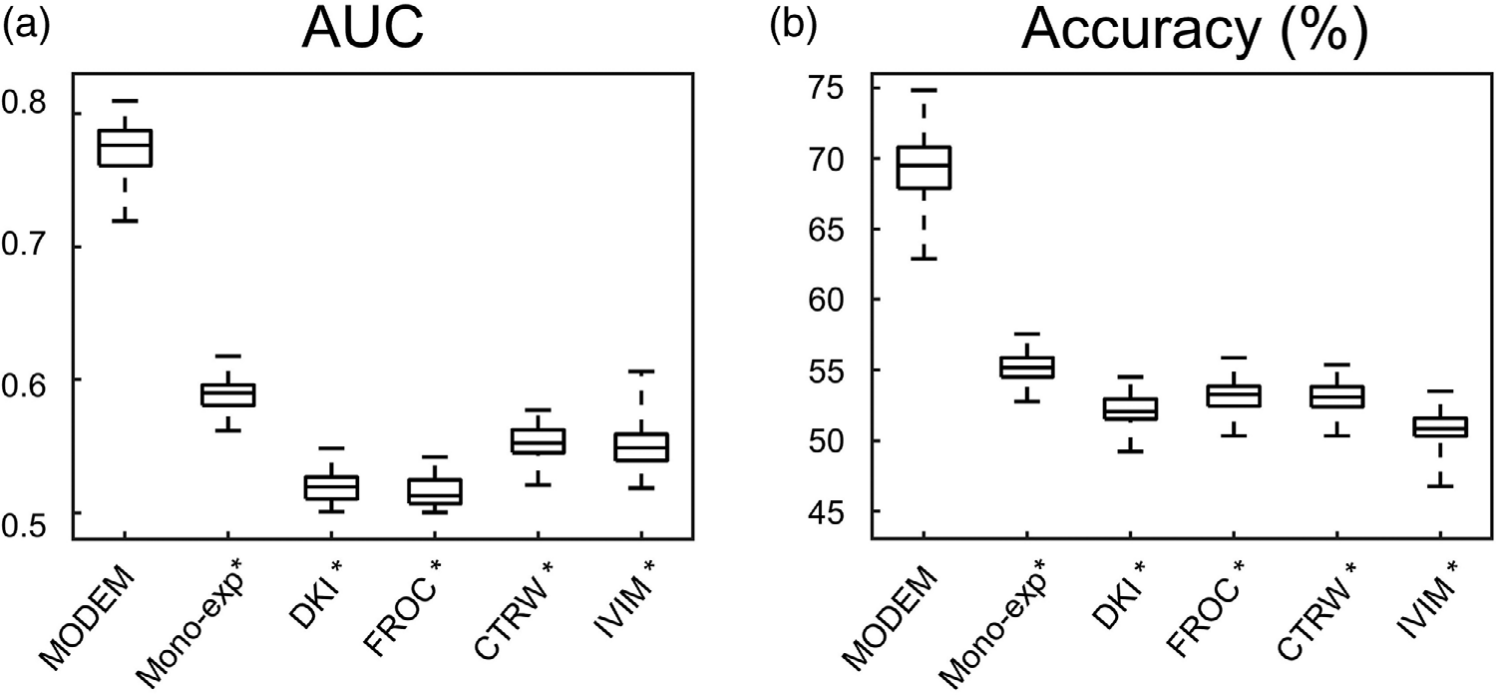
Boxplots of the AUC (a) and accuracy (b) of MODEM and the diffusion models for differentiating late‐stage from early‐stage cervical cancer over 100 iterations. *Indicates statistically significant difference with between MODEM and a specific diffusion model listed along the horizontal axis using *p* < 0.05 as a threshold. AUC, area under the curve; MODEM, MOdel‐free Diffusion‐wEighted MRI.

Figure 6 shows the color‐coded predicted probability maps from MODEM (with one iteration) and the five diffusion models, where each probability map is overlaid on DW images of an ROI from a patient with early‐stage cervical cancer (Figure 6a) or an ROI from another patient with late‐state cervical cancer (Figure 6b). In Figure 6a (early‐stage cervical cancer), MODEM indicated a lower probability of being classified as late‐stage cervical cancer than any of the diffusion models (MODEM, 0.389 ± 0.141; mono‐exponential, 0.481 ± 0.014; DKI, 0.505 ± 0.007; FROC, 0.512 ± 0.021; CTRW, 0.478 ± 0.025; IVIM, 0.498 ± 0.028). In Figure 6b (late‐stage cervical cancer), MODEM showed a substantially higher probability of being classified as late‐stage cervical cancer than any of the diffusion models (MODEM, 0.936 ± 0.114; mono‐exponential, 0.524 ± 0.011; DKI, 0.490 ± 0.006; FROC, 0.462 ± 0.014; CTRW, 0.480 ± 0.027; IVIM, 0.489 ± 0.024). Figure 7 displays the boxplots of AUC and accuracy of MODEM (with 100 iterations) and the diffusion models in differentiating early‐stage versus late‐stage cervical cancer from all 54 patients. Similar to the results of normal versus cancerous tissue differentiation shown in Figure 5, MODEM outperformed any of the diffusion models with statistical significance (*p* < 0.05), yielding the highest mean AUC (0.773 in MODEM vs. 0.589 in mono‐exponential, 0.520 in DKI, 0.516 in FROC, 0.553 in CTRW, and 0.549 in IVIM) and the highest accuracy (69.2% in MODEM vs. 55.2% in mono‐exponential, 52.1% in DKI, 53.1% in FROC, 53.2% in CTRW, and 50.8% in IVIM). Similar to cervical cancer detection, Table 2 shows that the diffusion coefficient remains the most influential parameter in cervical cancer staging, exhibiting the highest normalized logistic regression coefficient across all models.

## 4 DISCUSSION

In this study, we introduced a novel ML‐based, model free approach—MODEM—to improve tissue characterization by using a deep neural network with DWI signal intensities as input. Our results demonstrated that MODEM improved differentiation of tissue types over the conventional approach relying on analytical diffusion models, including mono‐exponential, DKI, FROC, CTRW, and IVIM models. Such improvements were observed not only in synthetic data generated with Monte‐Carlo simulations mimicking diverse tissue properties, but also in clinical data obtained from cervical cancer patients. In particular, MODEM exhibited significantly higher accuracy and AUC than any of the diffusion models in detecting and staging cervical cancer, underscoring its clinical potential.

To overcome the limitations of predefined assumptions and parameter degeneracy in traditional model‐based approach, ML‐based, model‐free methods have recently been investigated in the context of DWI data analysis. Ciritsis et al.^19^ utilized a k‐nearest neighbor (kNN) algorithm to distinguish abdominal organs using multi‐*b*‐value DWI data. Otsuka et al.^20^ applied a k‐means clustering method to multi‐*b*‐value DWI data for precise characterization of the inferior alveolar nerve. Both studies showcased the importance of leveraging ML‐based approach to characterize tissue properties without analytical modeling and parameter fitting. In this study, we extended the similar approach to diffusion MRI with a comprehensive set of *b*‐values and explored the potential applications to cervical cancer detection and staging, representing a significant step forward in leveraging model‐free techniques for classifying pathologically confirmed cancerous tissue.

Our simulation results demonstrated superior performance of MODEM in comparison to traditional model‐based DWI data analysis techniques. Across various substrates, MODEM consistently exhibited higher AUC and accuracy, particularly in scenarios where the noise levels exceeded 5%, which are typically encountered clinically. At a noise level of ≥ 10%, MODEM outperformed all diffusion models investigated, highlighting its robustness under practical situations. Additionally, the observed stability of MODEM's performance metrics, with SDs below 1% in both accuracy and AUC, further enhances its potential as an attractive tool for tissue characterization. More importantly, in the clinical demonstrations on patients with cervical cancer, MODEM markedly enhanced diagnostic capabilities and exhibited superior overall diagnostic performance to analytical diffusion models. For example, we observed a 5.1%–9.0% improvement in AUC and 5.9%–11.3% in accuracy for cervical cancer detection. For cervical cancer staging, the improvements were even more substantial (i.e., 23.5%–33.3% in AUC and 19.8%–26.1% in accuracy).

Unlike analytical diffusion models that rely on an iterative fitting process with predefined assumptions about tissue microstructures, MODEM is a data‐driven and model‐free approach that directly learns patterns from the DWI signal intensities. This distinction offers at least two advantages. First, by directly learning patterns from the DWI signal intensities at various *b*‐values without the intermediate fitting process, MODEM mitigates the issue of degeneracy, where multiple parameter combinations can fit to the data. Second, the deep neural network employed in MODEM can effectively extract high‐dimensional features from the DWI data, capturing subtle differences in signal attenuation patterns that may not be captured by traditional models. The model‐free nature of MODEM avoids the potential biases and limitations inherent in preselected diffusion models, particularly when dealing with complex tissues like tumors where the underlying microstructure and heterogeneity^31^, ^32^, ^33^ may deviate from simple assumptions.

It is important to note that traditional diffusion models remain valuable for understanding tissue microstructure and providing specific parameters, particularly when these parameters can be related to tissue biological or biophysical properties. For example, the mono‐exponential model provides information on water diffusivity in the underlying tissue and has been related to tissue cellularity.^34^, ^35^ Similarly, the IVIM model separates the DWI signal into distinct components reflecting perfusion (blood flow) and diffusion (molecular motion), aiding in the assessment of tumor vascularity.^4^ Other diffusion models have been developed to probe specific tissue properties,^5^, ^6^, ^8^, ^11^, ^12^, ^36^, ^37^ providing parameters sensitive to tissue properties such as diffusion barriers, tortuosity, cell size, compartmentalization, and heterogeneity.

Our study has several limitations. First, the use of simulated data, while valuable for gaining insights under a well‐controlled condition, cannot fully represent the complexity of actual biological tissues. Therefore, additional validation of MODEM on real‐world datasets from a broader range of tissue types and pathological states is warranted. Second, the current implementation of MODEM utilizes deep neural networks, making it challenging to relate its internal representations to specific tissue properties. Future research could explore the use of explainable artificial intelligence techniques, such as saliency maps^38^ or feature attribution methods,^39^ to identify the key features of the input diffusion signals contributing to the network's prediction. Third, our clinical validation relied on FIGO staging as the gold standard, which itself incorporates radiologists' assessment of DWI and ADC maps along with other imaging sequences. This approach may not serve as an independent reference since MODEM also uses DWI as input. Future studies could overcome this limitation by incorporating independent biomarkers or molecular markers from pathological samples as additional validation metrics, providing a better ground truth for assessing MODEM's performance. Finally, despite using each voxel as an independent data point, the size of the training data remains relatively small (19 180 for cervical cancer detection and 9835 for cervical cancer staging). Increasing the dataset size would enable the construction of a larger neural network in MODEM, enhancing its capacity to better reveal the intricate relationships within the data and facilitate a more nuanced representation of diverse tissue microstructures.

In conclusion, we have introduced MODEM—a novel ML‐based approach for tissue characterization based on raw diffusion‐weighted MRI data. By using simulation data and clinical cervical cancer diffusion‐weighted images, our study has shown that MODEM offers considerable advantages over the traditional methods with analytical diffusion models, particularly in improving the accuracy of cervical cancer detection and staging. Our demonstration also suggests that the MODEM approach may be extended to other applications, including tissue characterization during disease progression and regression, image segmentation, and beyond.

## CONFLICT OF INTEREST STATEMENT

X. J. Z. was a consultant of GE HealthCare prior to the start of this study. None of the other authors have any financial or other relationships that may cause, or are perceived to cause, conflict of interest in relation to the study during the period when the study was conducted.

## Data Availability

The code used in this study will be made available at https://github.com/uic‐cmr‐3t/MODEM from the date of publication. Other relevant image data sets will be released upon request to the corresponding author, subject to local Institutional Review Board and other regulations. Data sharing, including both software and image data, will conform to the relevant NIH policies and procedures.

## References

[1] Bernstein MA , King KF , Zhou XJ . Handbook of MRI Pulse Sequences. Elsevier; 2004.

[2] Le Bihan D , Poupon C , Amadon A , Lethimonnier F . Artifacts and pitfalls in diffusion MRI. J Magn Reson Imaging. 2006;24(3):478‐488. doi:10.1002/jmri.20683 16897692

[3] Le Bihan D , Breton E , Lallemand D , Grenier P , Cabanis E , Laval‐Jeantet M . MR imaging of intravoxel incoherent motions: application to diffusion and perfusion in neurologic disorders. Radiology. 1986;161(2):401‐407. 3763909 10.1148/radiology.161.2.3763909

[4] Iima M , Le Bihan D . Clinical intravoxel incoherent motion and diffusion MR imaging: past, present, and future. Radiology. 2016;278(1):13‐32. doi:10.1148/radiol.2015150244 26690990

[5] Jensen JH , Helpern JA , Ramani A , Lu H , Kaczynski K . Diffusional kurtosis imaging: the quantification of non‐Gaussian water diffusion by means of magnetic resonance imaging. Magn Reson Med. 2005;53(6):1432‐1440. doi:10.1002/mrm.20508 15906300

[6] Westin CF , Knutsson H , Pasternak O , et al. Q‐space trajectory imaging for multidimensional diffusion MRI of the human brain. Neuroimage. 2016;135:345‐362. doi:10.1016/j.neuroimage.2016.02.039 26923372 PMC4916005

[7] White NS , Leergaard TB , D'Arceuil H , Bjaalie JG , Dale AM . Probing tissue microstructure with restriction spectrum imaging: histological and theoretical validation. Hum Brain Mapp. 2013;34(2):327‐346. doi:10.1002/hbm.21454 23169482 PMC3538903

[8] Magin RL , Abdullah O , Baleanu D , Zhou XJ . Anomalous diffusion expressed through fractional order differential operators in the Bloch‐Torrey equation. J Magn Reson. 2008;190(2):255‐270. doi:10.1016/j.jmr.2007.11.007 18065249

[9] Zhou XJ , Gao Q , Abdullah O , Magin RL . Studies of anomalous diffusion in the human brain using fractional order calculus. Magn Reson Med. 2010;63(3):562‐569. doi:10.1002/mrm.22285 20187164

[10] Li Z , Dan G , Tammana V , et al. Predicting the aggressiveness of peripheral zone prostate cancer using a fractional order calculus diffusion model. Eur J Radiol. 2021;143:109913. doi:10.1016/j.ejrad.2021.109913 34464907

[11] Feng C , Wang Y , Dan G , et al. Evaluation of a fractional‐order calculus diffusion model and bi‐parametric VI‐RADS for staging and grading bladder urothelial carcinoma. Eur Radiol. 2022;32(2):890‐900. doi:10.1007/s00330-021-08203-2 34342693

[12] Karaman MM , Sui Y , Wang H , Magin RL , Li Y , Zhou XJ . Differentiating low‐ and high‐grade pediatric brain tumors using a continuous‐time random‐walk diffusion model at high b‐values. Magn Reson Med. 2016;76(4):1149‐1157. doi:10.1002/mrm.26012 26519663 PMC4852163

[13] Ingo C , Magin RL , Parrish TB . New insights into the fractional order diffusion equation using entropy and kurtosis. Entropy. 2014;16(11):5838‐5852. doi:10.3390/e16115838 28344436 PMC5365032

[14] Hall MG , Ingo C . Half way there: theoretical considerations for power laws and sticks in diffusion MRI for tissue microstructure. Mathematics. 2021;9(16):1871. doi:10.3390/math9161871

[15] Harms RL , Fritz FJ , Tobisch A , Goebel R , Roebroeck A . Robust and fast nonlinear optimization of diffusion MRI microstructure models. Neuroimage. 2017;155:82‐96. doi:10.1016/j.neuroimage.2017.04.064 28457975 PMC5518773

[16] Gustafsson O , Montelius M , Starck G , Ljungberg M . Impact of prior distributions and central tendency measures on Bayesian intravoxel incoherent motion model fitting. Magn Reson Med. 2018;79(3):1674‐1683. doi:10.1002/mrm.26783 28626964

[17] Jelescu IO , Veraart J , Fieremans E , Novikov DS . Degeneracy in model parameter estimation for multi‐compartmental diffusion in neuronal tissue. NMR Biomed. 2016;29(1):33‐47. doi:10.1002/nbm.3450 26615981 PMC4920129

[18] Golkov V , Dosovitskiy A , Sperl JI , et al. q‐Space deep learning: twelve‐fold shorter and model‐free diffusion MRI scans. IEEE Trans Med Imaging. 2016;35(5):1344‐1351. doi:10.1109/TMI.2016.2551324 27071165

[19] Ciritsis A , Rossi C , Wurnig MC , Phi Van V , Boss A . Intravoxel incoherent motion: model‐free determination of tissue type in abdominal organs using machine learning. Invest Radiol. 2017;52(12):747‐757. doi:10.1097/RLI.0000000000000400 28742733

[20] Otsuka A , Terumitsu M , Matsuzawa H , Watanabe M , Seo K . Model‐free cluster analysis for multi‐b‐value diffusion‐weighted imaging of the inferior alveolar nerve. J Oral Maxillofac Radiol. 2023;11(1):16. doi:10.4103/jomr.jomr_2_23

[21] Ciritsis A , Boss A , Rossi C . Automated pixel‐wise brain tissue segmentation of diffusion‐weighted images via machine learning. NMR Biomed. 2018;31(7):e3931. doi:10.1002/nbm.3931 29697165

[22] Troelstra MA , Van Dijk AM , Witjes JJ , et al. Self‐supervised neural network improves tri‐exponential intravoxel incoherent motion model fitting compared to least‐squares fitting in non‐alcoholic fatty liver disease. Front Physiol. 2022;13:942495. doi:10.3389/fphys.2022.942495 PMC9485997 36148303

[23] Barbieri S , Gurney‐Champion OJ , Klaassen R , Thoeny HC . Deep learning how to fit an intravoxel incoherent motion model to diffusion‐weighted MRI. Magn Reson Med. 2020;83(1):312‐321. doi:10.1002/mrm.27910 31389081

[24] Kaandorp MPT , Barbieri S , Klaassen R , et al. Improved unsupervised physics‐informed deep learning for intravoxel incoherent motion modeling and evaluation in pancreatic cancer patients. Magn Reson Med. 2021;86:2250‐2265. 34105184 10.1002/mrm.28852 PMC8362093

[25] Huang Y , Bert C , Sommer P , et al. Deep learning for brain metastasis detection and segmentation in longitudinal MRI data. Med Phys. 2022;49(9):5773‐5786. doi:10.1002/mp.15863 35833351

[26] Mastropietro A , Procissi D , Scalco E , Rizzo G , Bertolino N . A supervised deep neural network approach with standardized targets for enhanced accuracy of IVIM parameter estimation from multi‐SNR images. NMR Biomed. 2022;35(10):e4774. doi:10.1002/nbm.4774 PMC9539583 35587618

[27] Cook PA , Bai Y , Hall MG , Nedjati‐Gilani S , Seunarine KK , Alexander DC . Camino: diffusion MRI reconstruction and processing. Int Soc Magn Reson Med Seattle. 2006: 2759. http://www.insight‐journal.com/download/pdf/104/camino.pdf

[28] Hall MG , Alexander DC . Convergence and parameter choice for Monte‐Carlo simulations of diffusion MRI. IEEE Trans Med Imaging. 2009;28(9):1354‐1364. doi:10.1109/TMI.2009.2015756 19273001

[29] Ingo C , Magin RL , Colon‐Perez L , Triplett W , Mareci TH . On random walks and entropy in diffusion‐weighted magnetic resonance imaging studies of neural tissue. Magn Reson Med. 2014;71(2):617‐627. doi:10.1002/mrm.24706 23508765 PMC4930657

[30] Qi LP , Yan WP , Chen KN , et al. Discrimination of malignant versus benign mediastinal lymph nodes using diffusion MRI with an IVIM model. Eur Radiol. 2018;28(3):1301‐1309. doi:10.1007/s00330-017-5049-8 28929210

[31] Tang L , Sui Y , Zhong Z , et al. Non‐Gaussian diffusion imaging with a fractional order calculus model to predict response of gastrointestinal stromal tumor to second‐line sunitinib therapy. Magn Reson Med. 2018;79(3):1399‐1406. doi:10.1002/mrm.26798 28643387 PMC5741547

[32] Karaman MM , Zhou CY , Zhang J , Zhong Z , Wang K , Zhu W . Percentile‐based analysis of non‐gaussian diffusion parameters for improved glioma grading. Investig Magn Reson Imaging. 2022;26(2):104‐116. doi:10.13104/imri.2022.26.2.104

[33] Karaman MM , Zhang J , Xie KL , Zhu W , Zhou XJ . Quartile histogram assessment of glioma malignancy using high b‐value diffusion MRI with a continuous‐time random‐walk model. NMR Biomed. 2021;34(4):e4485. doi:10.1002/nbm.4485 33543512

[34] Tang L , Zhou XJ . Diffusion MRI of cancer: from low to high b‐values. J Magn Reson Imaging. 2019;49(1):23‐40. doi:10.1002/jmri.26293 30311988 PMC6298843

[35] Le Bihan D . The “wet mind”: water and functional neuroimaging. Phys Med Biol. 2007;52(7). doi:10.1088/0031-9155/52/7/R02 17374909

[36] Baron CA , Beaulieu C . Oscillating gradient spin‐echo (OGSE) diffusion tensor imaging of the human brain. Magn Reson Med. 2014;72(3):726‐736. doi:10.1002/mrm.24987 24142863

[37] Xu J , Jiang X , Devan SP , et al. MRI‐cytometry: mapping nonparametric cell size distributions using diffusion MRI. Magn Reson Med. 2021;85(2):748‐761. doi:10.1002/mrm.28454 32936478 PMC7722100

[38] Simonyan K , Vedaldi A , Zisserman A . Deep inside convolutional networks: visualising image classification models and saliency maps. In: International Conference on Learning Representations (ICLR) 2014 ‐ Work Track Proc . 2014:1‐8.

[39] Ribeiro MT , Singh S , Guestrin C . “Why should i trust you?” Explaining the predictions of any classifier. In: Proceedings of the 22nd ACM SIGKDD International Conference on Knowledge Discovery and Data Mining. ACM; 2016:1135‐1144. doi:10.1145/2939672.2939778

